# SUPPORTING FORTITUDE AMONG FAMILY CAREGIVERS: THE ROLE OF ADULT DAY SERVICES FOR PERSONS WITH DEMENTIA

**DOI:** 10.1093/geroni/igae098.0771

**Published:** 2024-12-31

**Authors:** Heather Young, Orly Tonkikh, Everlyne Ogugu, Jessica Famula, Janice Bell

**Affiliations:** UC Davis, Ashland, Oregon, United States; University of California Davis, Davis, California, United States; University of California Davis, Davis, California, United States; University of California Davis, Davis, California, United States; UC Davis, Davis, California, United States

## Abstract

Caring for a person with dementia requires family fortitude in terms of commitment, time, energy and ability to manage a trajectory of several years. Family caregivers provide the vast majority of care for over 7 million individuals living with dementia. Many are adult children who deal with competing demands of other family members and employment. Adult Day Services (ADS) offer respite for family caregivers and meaningful support for persons with dementia. This study presents caregiver perspectives from the California Community Program for Alzheimer’s Supports and Services Pilot Program that engaged seven sites in a learning community to develop care standards and advance equity. We conducted semi-structured interviews and thematic analysis of recorded transcripts. The sample included 24 caregivers aged 25 to over 85 (12 spouses, 10 adult children and 2 other relatives). Caregivers described ADS services as life sustaining, being a vital resource that enabled them to continue in their role, balancing other demands. ADS improved mental health and physical health, reduced strain, and fostered self-care and engagement in the broader social network. The services also provided education and enhanced their skills in caring, prolonging their ability to continue. ADS services benefit both persons with dementia and caregivers. Future research could illuminate the long-range health care and societal costs and benefits of ADS for both persons with dementia and caregivers. Sustainable solutions bolster fortitude and optimize person and family-centered care.

